# Online Newspaper Reports on Ambulance Accidents in Austria, Germany, and Switzerland: Retrospective Cross-sectional Review

**DOI:** 10.2196/25897

**Published:** 2021-11-12

**Authors:** Johanna Boldt, Femke Steinfort, Martin Müller, Aristomenis K Exadaktylos, Jolanta Klukowska-Roetzler

**Affiliations:** 1 Department of Emergency Medicine Inselspital, Bern University Hospital Bern University Berne Switzerland

**Keywords:** ambulance accidents, ambulance collisions, ambulance crashes, media-based, media-based review, newspaper review, Austria, Germany, Switzerland, German-speaking European countries, retrospective, cross-sectional, review, ambulance, accident, data, media, newspaper

## Abstract

**Background:**

Ambulance accidents are an unfortunate indirect result of ambulance emergency calls, which create hazardous environments for personnel, patients, and bystanders. However, in European German-speaking countries, factors contributing to ambulance accidents have not been optimally researched and analyzed.

**Objective:**

The objective of this study was to extract, analyze, and compare data from online newspaper articles on ambulance accidents for Austria, Germany, and Switzerland. We hope to highlight future strategies to offset the deficit in research data and official registers for prevention of ambulance and emergency vehicle accidents.

**Methods:**

Ambulance accident data were collected from Austrian, German, and Swiss free web-based daily newspapers, as listed in Wikipedia, for the period between January 2014 and January 2019. All included newspapers were searched for articles reporting ambulance accidents using German terms representing “ambulance” and “ambulance accident.” Characteristics of the accidents were compiled and analyzed. Only ground ambulance accidents were covered.

**Results:**

In Germany, a total of 597 ambulance accidents were recorded, corresponding to 0.719 (95% CI 0.663-0.779) per 100,000 inhabitants; 453 of these accidents left 1170 people injured, corresponding to 1.409 (95% CI 1.330-1.492) per 100,000 inhabitants, and 28 of these accidents caused 31 fatalities, corresponding to 0.037 (95% CI 0.025-0.053) per 100,000 inhabitants. In Austria, a total of 62 ambulance accidents were recorded, corresponding to 0.698 (95% CI 0.535-0.894) per 100,000 inhabitants; 47 of these accidents left 115 people injured, corresponding to 1.294 (95% CI 1.068-1.553) per 100,000 inhabitants, and 6 of these accidents caused 7 fatalities, corresponding to 0.079 (95% CI 0.032-0.162) per 100,000 inhabitants. In Switzerland, a total of 25 ambulance accidents were recorded, corresponding to 0.293 (95% CI 0.189-0.432) per 100,000 inhabitants; 11 of these accidents left 18 people injured, corresponding to 0.211(95% CI 0.113-0.308) per 100,000 inhabitants. There were no fatalities. In each of the three countries, the majority of the accidents involved another car (77%-81%). In Germany and Switzerland, most accidents occurred at an intersection. In Germany, Austria, and Switzerland, 38.7%, 26%, and 4%, respectively, of ambulance accidents occurred at intersections for which the ambulance had a red light (*P*<.001). In all three countries, most of the casualties were staff and not uncommonly a third party. Most accidents took place on weekdays and during the daytime. Ambulance accidents were evenly distributed across the four seasons. The direction of travel was reported in 28%-37% of the accidents and the patient was in the ambulance approximately 50% of the time in all countries. The cause of the ambulance accidents was reported to be the ambulance itself in 125 (48.1% of accidents where the cause was reported), 22 (42%), and 8 (40%) accidents in Germany, Austria, and Switzerland, respectively (*P*=.02), and another vehicle in 118 (45.4%), 29 (56%), and 9 (45%) accidents, respectively (*P*<.001). A total of 292 accidents occurred while blue lights and sirens were used, which caused 3 deaths and 577 injuries.

**Conclusions:**

This study draws attention to much needed auxiliary sources of data that may allow for creation of a contemporary registry of all ambulance accidents in Austria, Germany, and Switzerland. To improve risk management and set European standards, it should be mandatory to collect standardized goal-directed and representative information using various sources (including the wide range presented by the press and social media), which should then be made available for audit, analysis, and research.

## Introduction

**Figure 1:**
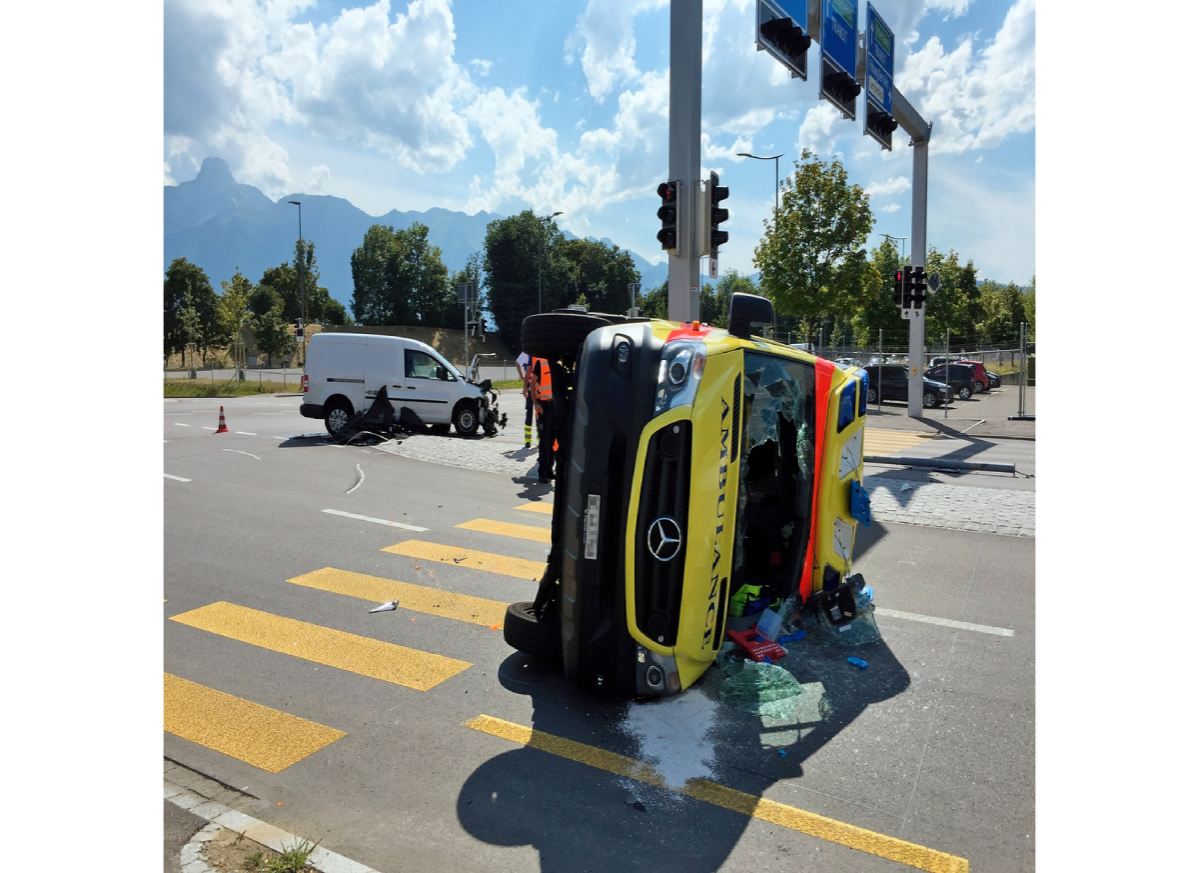
Collision of a delivery van with an ambulance using blue lights and sirens on the way to a patient in Thun, Switzerland. Photo: Michael Gurtner.

Ambulances respond to medical emergencies worldwide. Such emergencies often include hazardous processes and environments for the personnel, patients, and bystanders [1,2]. Unfortunately, ambulances can also be involved in accidents, leading to additional injured people and even fatalities. A representative example is an accident in Thun, Switzerland, where a delivery van collided with an ambulance using blue lights and sirens on the way to a patient. This must have been a high-impact collision at the intersection, because the ambulance overturned and skidded to a standstill. Two ambulance staff members and the delivery van driver were injured. Another two ambulances brought the three injured individuals to hospital for evaluation. The police reported considerable disruption due to the central location of the accident site. The surrounding roads were temporarily closed and traffic was diverted. Many police patrols, the fire brigade, the military, the Federal Roads Office, and a tow truck were present at the scene of the accident (Figure 1) [3].

Austria, Germany, and Switzerland have comparable organizational structures and response systems for ambulance services, which are provided by multiple emergency service providers in each city, municipality, or region. Austrian Ambulance Emergency Services (AAES) are organized individually by each of the nine federal states and by some cities (Vienna and Graz). The AAES is efficiently coordinated between private organizations and other providers in each area. Germany also organizes emergency ambulance and health services internally in each of the 16 federal states via more than one public and/or private provider of emergency services.

In Switzerland, the responsibility and organization of emergency ambulance response services rest with each canton and some individual municipalities. Switzerland stores the information on all emergency service accidents (police, fire brigade, and other) in central databases, which makes it difficult to analyze the data for ambulances alone, thereby hindering data analysis to improve the safety of patients, passengers, and the public. The lack of appropriate data from the responsible institutions is not a unique problem. For example, Chester et al [4] experienced similar difficulties when trying to obtain helicopter accident data from 1987 to 2014 for the United Kingdom, Germany, and the United States. Certain states in the United States and other parts of the world have systems in place for data collection and analysis of ambulance accidents/crashes [5-8]. Nevertheless, Sanddal et al [9] found that these data are often not accessible for further research. In Austria, Germany, and Switzerland, published research on ambulance accidents is very limited. Given that so little has been published for each individual country, an alternative information source needs to be explored. This study was therefore based on analysis of media reports, which positions itself as the most accessible and relevant source.

For many years, various media types have become a major source of information for the vast majority of the population. The media provide significant news coverage that is of public interest. The first weekly newspaper in the world was printed in 1604 in Strasbourg, which was then part of the Holy Roman Empire. In the early 1900s, a retired mechanical engineer, Hugh DeHaven, collected data from newspapers, magazines, and journals on the mechanisms of death and injuries due to airplanes, as well as human and vehicular accidents. This was a remarkable accomplishment, since he had received no institutional support. DeHaven is thus considered to be the “father of crashworthiness research.” Together with medical institutions, he encouraged and lobbied for crash injury studies aiming to increase the safety of aircrafts and cars by providing relevant information to production engineers and manufacturers [10]. Media-based research is increasingly used as an information source, including selfie-related deaths, which would only be reported in the media [11-13]. Woodcock [14] further studied the contributing factors to amusement park ride accidents, as reported by the media. Sandal et al [9] extended their initial peer-reviewed publication to identify the factors involved in rural ambulance accidents by including the popular press for more descriptive data. Since 1997, medical internet research as an entity has been represented and published electronically in a dedicated peer-review online journal [15]. Reporters such as Herrnkind have indicated that ambulance collisions may not be so uncommon; for example, in 2017, the Stern newspaper commented “Every few days it crashes” [16,17].

We failed to find published reviews on the outcome and factors involved in ambulance accidents in Austria, Germany, and Switzerland. To our knowledge, no research has compared ambulance accidents in Austria, Germany, and Switzerland, nor have the media been used as a data source to investigate these incidents. Information in daily web-based free newspapers is easily accessible and is of interest to researchers, the public, and the local press. This review is therefore a retrospective study of web-based newspaper articles reporting ambulance accidents in the main three German-speaking European countries for the period between January 2014 and January 2019. The objective was to extract, analyze, and compare data from online newspaper articles on ambulance accidents for these countries. We hope to highlight the deficit in research data and official registers relevant for the prevention of future accidents with ambulance and emergency vehicles. Extractable and comparable data would go a long way toward identifying the cause of ambulance accidents. In turn, this may allow additional protocols to be implemented for the prevention of future ambulance and emergency vehicle accidents.

## Methods

Data were collected from articles in web-based daily free newspapers, as listed in Wikipedia for Germany, Austria, and Switzerland [18-20]. All included newspapers were searched for articles reporting ambulance accidents in the three countries between January 2014 and January 2019, using the following German search terms: “Ambulanz,” “Rettungswagen,” “Rettungsauto,” “Krankenwagen,” “Unfall Ambulanz,” “Unfall Rettungswagen,” “Unfall Rettungsauto,” and “Unfall Krankenwagen.” These terms represent “ambulance” and “ambulance accident” in the English language. In Switzerland, the search of French, Italian, and Rhaeto-Romansh newspapers was performed in the relevant languages. If accidents were reported in multiple newspapers, the article that provided the most data was used or data were extracted from more than one article when the data were cumulative. Some accidents involving ambulances were reported in newspapers requiring a subscription fee to access the relevant article; these were excluded unless such incidents were published in free online newspapers. A considerable number of listed newspapers are managed by the same publisher and rerouted to identical websites. The research data were obtained from a total of 203 daily online newspapers. Only ground ambulance accidents were covered in this study.

Data compiled included the number of accidents reported, number of people transported by the ambulance, number of people involved in the accident (including other vehicles and/or pedestrians), outcome of the people involved in the accident (injury or death), environmental demographics, destination of the ambulance, possible use of blue light and/or siren, people involved in the ambulance accident (staff, patients, or bystanders), date, day of the week, time of day, place/type of road/type of intersection, traffic signals, and the cause of the accident. Data that were not available in a newspaper article are described as “unknown.”

The above characteristics were analyzed using Microsoft Excel for Mac (Version 16.47, Microsoft Corporation) and Stata 16.1 (StataCorp). Associations of categorical variables and countries were evaluated using the χ^2^ test. Continuous variables between countries were compared using the Kruskal-Wallis test.

## Results

Extensive data were gathered from online newspapers between January 2014 and January 2019 (Table 1).

**Table 1:** Ambulance accidents in Germany, Austria, and Switzerland.

State/canton		Ambulance accidents, n (%)^a^	Inhabitants in 2018, n	Accidents/100,000 inhabitants (95% CI)
**Germany**				
	Baden Wurttemberg	105 (17.6)	11,069,533	0.949 (0.767 to 1.130)
	Bavaria	144 (24.1)	13,076,721	1.101 (0.921 to 1.281)
	Berlin	38 (6.4)	3,644,826	1.043 (0.711 to 1.374)
	Brandenburg	9 (1.5)	2,511,917	0.358 (0.124 to 0.592)
	Bremen	7 (1.2)	682,986	1.025 (0.266 to 1.784)
	Hamburg	11 (1.8)	1,841,179	0.597 (0.244 to 0.555)
	Hesse	25 (4.2)	6,265,809	0.399 (0.243 to 0.555)
	Lower Saxony	45 (7.5)	7,982,448	0.564 (0.399 to 0.728)
	Mecklenburg-Western Pomerania	10 (1.7)	1,609,675	0.621 (0.236 to 1.006)
	North Rhine-Westphalia	120 (20.1)	17,932,651	0.669 (0.549 to 0.789)
	Rhineland-Palatinate	16 (2.7)	4,084,844	0.392 (0.200 to 0.584)
	Saarland	10 (1.7)	990,509	1.010 (0.384 to 1.635)
	Saxony	18 (3.0)	4,077,937	0.441 (0.237 to 0.645)
	Saxony-Anhalt	22 (3.7)	2,208,321	0.996 (0.580 to 1.413)
	Schleswig-Holstein	14 (2.4)	2,896,712	0.483 (0.230 to 0.736)
	Thuringia	3 (0.5)	2,143,145	0.140 (0.000 to 0.298)
	Total	597 (100.0)	83,019,213 [21]^b^	0.719 (0.661 to 0.777)
	Total accidents causing injury	453 (75.9)	83,019,213	0.546 (0.495 to 0.596)
	Total accidents causing death	28 (4.7)	83,019,213	0.034 (0.021 to 0.046)
**Austria**				
	Burgenland	2 (3)	292,675	0.683 (0.000 to 1.630)
	Carinthia	8 (13)	560,898	1.426 (0.438 to 2.415)
	Lower Austria	5 (8)	1,670,668	0.299 (0.037 to 0.562)
	Salzburg	3 (5)	552,579	0.543 (0.000 to 1.157)
	Styria	14 (23)	1,240,214	1.129 (0.538 to 1.720)
	Tyrol	5 (8)	75,114	6.657 (0.822 to 12.491)
	Upper Austria	15 (24)	1,473,576	1.018 (0.503 to 1.533)
	Vienna	4 (6)	1,888,776	0.212 (0.004 to 2.757)
	Vorarlberg	6 (10)	391,741	1.532 (0.306 to 2.757)
	Total	62 (100)	8,888,775 [22]^b^	0.698 (0.524 to 0.871)
	Total accidents causing injury	47 (76)	8,888,775	0.529 (0.378 to 0.680)
	Total accidents causing death	6 (10)	8,888,775	0.068 (0.013 to 0.122)
**Switzerland**				
	Appenzell Outer-Rhodes	2 (8)	55,234	3.621 (0.000 to 8.639)
	Basel-Country	1 (4)	287,032	0.348 (0.000 to 1.031)
	Berne	5 (20)	1,034,977	0.483 (0.060 to 0.907)
	Grisons	1 (4)	198,379	0.504 (0.000 to 1.492)
	Lucerne	1 (4)	409,557	0.244 (0.000 to 0.723)
	Solothurn	1 (4)	273,194	0.366 (0.000 to 1.083)
	St Gallen	7 (28)	507,697	1.379 (0.357 to 2.400)
	Ticino	1 (4)	353,343	0.283 (0.000 to 0.838)
	Zug	3 (12)	126,837	2.365 (0.000 to 5.042)
	Zurich	3 (12)	1,520,968	0.197 (–0.026 to 0.420)
	Total	25 (100)	8,544,527 [23]^b^	0.293 (0.178 to 0.407)
	Total accidents causing injury	11 (44)	8,544,527	0.129 (0.053 to 0.205)
	Total accidents causing death	0 (0)	8,544,527	0.000 (0.000 to 0.000)

^a^Due to rounding, percentages may not add up to 100.

^b^References [21-23] represent the population of each country in 2018.

German newspapers reported a total of 597 ambulance accidents, corresponding to 0.719/100,000 inhabitants. In total, 453 of these accidents left 1170 people injured, corresponding to 1.409/100,000 inhabitants; 28 of these accidents caused 31 fatalities, corresponding to 0.037/100,000 inhabitants. Austrian newspapers reported a total of 62 ambulance accidents, corresponding to 0.698 /100,000 inhabitants. In total, 47 of these accidents left 115 people injured, corresponding to 1.294/100,000 inhabitants; 6 of these accidents caused 7 fatalities, corresponding to 0.079/100,000 inhabitants. Swiss newspapers reported a total of 25 ambulance accidents, corresponding to 0.293/100,000 inhabitants. A total of 11 of these accidents left 18 people injured, corresponding to 0.211/100,000 inhabitants. There were no fatalities (Table 1 and Table 2).

For each accident with an ambulance, on average two people were injured, except in Switzerland reporting a lower rate of 0.72 injured per accident. In all three countries, most of the casualties were staff and, not uncommonly, a third party. In Austria and Germany, ambulance accidents caused the death of a third party, patient, and staff in over 50%, 30%, and 13% of cases, respectively. Two children succumbed in these accidents in Germany. According to our data, the fatal ambulance accident incidence per 100,000 inhabitants was 0.034 for Germany, 0.068 for Austria, and zero for Switzerland (Table 2).

The newspaper articles also reported whether only blue light, sirens alone, or both blue light and sirens were used by the ambulance. The highest number of accidents with both blue light and sirens was reported in Germany, followed by Switzerland and Austria (*P*<.001). Blue light alone was reported to be used in 71 (12% of total accidents), 7 (11%), and 3 (12%) accidents in Germany, Austria, and Switzerland, respectively (*P*=.01). Sirens alone were recorded during one transport by ambulance in Germany and one in Austria; 291 ambulance accidents during which blue light and sirens were used caused 3 deaths and 573 injuries in total (Table 2). The absence of sirens or blue light was also mentioned in some newspaper accounts.

**Table 2:** Injuries and fatalities reported in Germany, Austria, and Switzerland.^a^

Accidents reported			Germany (n=597)	Austria (n=62)	Switzerland (n=25)	*P* value	
**Accidents causing injury**							
	Total, n (%)		453 (75.9)	47 (76)	11 (44)	<.001	
	**Injured (cumulative), n**		1170	115	18		
		Staff, n (%)	537 (45.9)	47 (41)	8 (44)		
		Third party, n (%)	217 (18.6)	8 (7)	8 (44)		
		Patient, n (%)	113 (9.7)	12 (10)	2 (11)		
		Children, n (%)	7 (0.6)	3 (3)	0 (0)		
		Not reported	296 (25.3)	45 (39)	0 (0)		
	Injured persons per accident, mean (95% CI)		1.96 (1.76-2.15)	1.85 (1.49-2.22)	0.72 (0.28-1.16)	<.001	
	Injured/100,000 inhabitants (95% CI)		1.409 (1.329-1.49)	1.294 (1.057-1.53)	0.211 (0.113-0.308)	<.001	
**Accidents causing death**							
	Total, n (%)		28 (4.7)	6 (10)	0 (0)	<.001	
	**Fatalities (cumulative), n**		31	7	0		
		Third party, n (%)	16 (51.6)	4 (67)	0 (0)		
		Patient in the ambulance, n (%)	9 (29.0)	1 (17)	0 (0)		
		Staff, n (%)	4 (12.9)	1 (17)	0 (0)		
		Children, n (%)	2 (6.5)	0 (0)	0 (0)		
	Fatalities per accident, mean (95% CI)		0.052 (0.032-0.072)	0.113 (0.020-0.201)	0	.74	
	Fatalities/100,000 citizens (95% CI)		0.037 (0.024-0.05)	0.079 (0.02-0.137)	0	<.001	
**Accidents causing death or injury (/100,000 inhabitants)**							
	Total, n (%)		461 (77.2)	49 (79.03)	11 (44.99)	<.001	
	Injured or death/100,000 inhabitants (95% CI)		0.555 (0.505-0.606)	0.551 (0.397-0.706)	0.129 (0.053-0.205)		
	Blue light and sirens, n (%)		272 (45.6)	8 (13)	10 (40)	<.001	
	Blue light and sirens causing death and injury, n (% of all blue light and sirens)		213 (77.6)	8 (100)	7 (70)	.27	

^a^Due to rounding, percentages may not add up to 100.

During the investigation period, the number of published ambulance accidents per year remained stable in all countries (*P*=.41)

The majority (77%-81%) of the accidents involved another car (Table 3). In Germany, most accidents occurred at an intersection, junction, or simply on a stretch of road in the city (street). In Austria, accidents most commonly occurred on the street, on a regional road, or at an intersection. For Switzerland, the highest accident incidence was on the street, followed by intersections and highways (Table 3).

Ambulance accidents on the street were further classified (Table 3). Most frequently, street accidents involved turning off or overtaking. For 143 of the 296 ambulance accidents that occurred at an intersection and one at a zebra crossing, the report included whether the traffic light was red or not. In Germany, Austria, and Switzerland, 38.8%, 27%, and 4% of ambulance accidents occurred at intersections for which the ambulance had the red light (*P*<.001). The red traffic light was disobeyed twice by other vehicles and once at a zebra crossing by a child, resulting in a fatality.

**Table 3:** Collision object, location, and location on the city street of ambulance accidents (N=684).

Accident details		Proportion of reported, n (%)^a^	Proportion of total accidents reported, %^a^
**Object the ambulance collided with (n=611)**			
	Passenger car	489 (80.0)	71.5
	Lorry	38 (6.2)	5.5
	Person	24 (3.9)	3.5
	Two-wheeler	18 (3.0)	2.6
	Service car	10 (1.5)	1.5
	Tram/train	9 (1.5)	1.3
	Object	8 (1.3)	1.2
	Bus	7 (1.2)	1.0
	Animal	6 (1.0)	0.9
	Tractor	2 (0.3)	0.3
**Location of the accident (n=649)**			
	Intersection	296 (45.6)	43.3
	Street	192 (29.6)	28.1
	City	49 (7.6)	7.2
	Regional road	38 (5.9)	5.6
	Highway	33 (5.1)	4.8
	Bend	25 (3.9)	3.7
	Accident site	6 (0.9)	0.9
	Other	5 (0.8)	0.7
	Railway	3 (0.5)	0.4
	Rescue lane	2 (0.3)	0.3
**Location on a street in the city (n=80)**			
	Turning off	45 (56)	6.58
	Overtaking	10 (13)	1.46
	Driveway	4 (5)	0.58
	Exit	4 (5)	0.58
	Lane change	4 (5)	0.58
	U-turn	2 (3)	0.29
	Rescue lane	2 (3)	0.29
	Road traffic light	2 (3)	0.29
	Zebra crossing	1 (1)	0.15
	Roadworks	1 (1)	0.15
	While crossing	1 (1)	0.15
	Congestion	1 (1)	0.15
	Turning in	1 (1)	0.15
	Narrowing	1 (1)	0.15
	Accident site	1 (1)	0.15

^a^Due to rounding, percentages do not add up to 100.

Ambulance accidents were evenly distributed over the four seasons. Most accidents took place on weekdays, with few accidents occurring at night (between 6:00 pm and 6:00 am). There was no significant difference between the incidence of ambulance accidents in the three countries with respect to seasons (*P*=.28), weekends (*P*=.56), or time of day (*P*=.64) (Table 4).

The direction of travel of the ambulance at the time of the accident was documented for 268/597 (44.9%), 29/62 (47%), and 9/25 (36%) accidents in Germany, Austria, and Switzerland, respectively (*P*=.64). Whether or not the patient was in the ambulance at the time of the accident was indicated in 54.8%, 74%, and 64% of reports for Germany, Austria, and Switzerland, respectively. Third-party violence caused injury to staff members in four cases in Germany and once for Austria and Switzerland each. In Switzerland, there was a report of one patient causing injury to a staff member (Table 4).

**Table 4:** Direction of travel, patient in ambulance, violence reported, day of the week, and time of day of the ambulance accidents in Germany, Austria, and Switzerland.

Characteristic of the accident		Germany, n (%)^a^	Austria, n (%)^a^	Switzerland, n (%)^a^	*P* value
**Direction of travel**					<.001
	Total reported cases, n	268	29	9	
	To the patient	154 (57.5)	18 (62)	6 (67)	
	To the hospital	114 (42.5)	9 (31)	3 (33)	
	Other	0 (0.0)	2 (7)	0 (0)	
**Patient in ambulance**					.79
	Total of reported cases, n	327	39	16	
	Yes	167 (51.1)	21 (54)	7 (44)	
	No	160 (48.9)	18 (46)	9 (56)	
**Violence toward staff**					<.001
	Total of reported cases, n	4	1	2	
	Third party	4 (1)	1 (2)	1 (4)	
	Patient	0 (0)	0 (0)	1 (4)	
**Part of the week**					.56
	Total of reported cases, n	597	62	25	
	Weekdays	458 (76.7)	50 (81)	21 (84)	
	Weekend	139 (23.3)	12 (19)	4 (16)	
**Time of day**					.64
	Total of reported cases, n	502	54	21	
	Day	369 (72.1)	38 (70)	17 (81)	
	Night	143 (27.9)	16 (30)	4 (19)	

^a^Due to rounding, percentages do not add up to 100.

**Figure 2:**
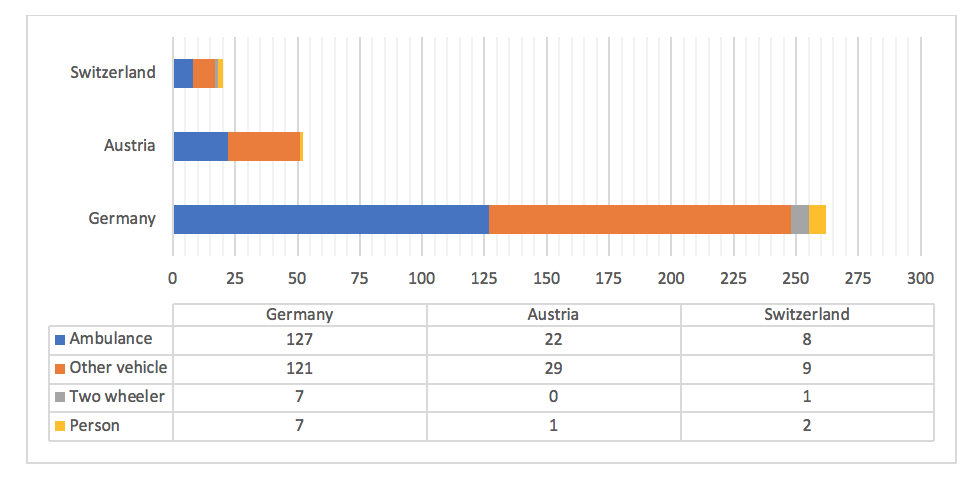
Cause of ambulance accidents in Austria, Germany, and Switzerland (*P*=.48).

The newspaper reports provided information to determine the cause of ambulance accidents in 43.5%, 83%, and 80% of total cases in Germany, Austria, and Switzerland, respectively (*P*<.001). The cause of the ambulance accidents was reported to be the ambulance itself in 125 accidents in Germany (representing 48.1% of accidents for which the cause was reported), 22 (42%) in Austria, and 8 (40%) in Switzerland (*P*=.52), and by another vehicle in 118 collisions (45.4%) in Germany, 29 (56%) in Austria, and 9 (45%) in Switzerland (*P*=.04) (Figure 2).

## Discussion

### Principal Findings

Currently, most countries provide emergency services for their citizens, such as police, fire, rescue, and medical services. Ambulances are equipped to transport patients to and from hospitals and provide initial care for medical and trauma emergencies. An ambulance is not designed for high-speed driving nor are most drivers trained for complicated decision-making during high-speed driving. Unfortunately, ambulance accidents are an element of these emergency services. Auerbach [24] reported that an ambulance accident delays the patient’s arrival in hospital by a mean of 9.4 minutes. Notwithstanding an overall decrease in road traffic accidents (RTAs) in the last 10 years, a slight rise has been observed since 2016. This downward trend may be explained (at least partially) by improvements in car safety design and increased use of bicycles. The recent rise may be associated with distractions such as the use of smartphones.

Switzerland exhibited a significantly lower rate of ambulance accidents than Germany and Austria (Table 1). Our data suggest that certain cantons in Switzerland were spared from ambulance accidents. This may be explained by the very low accident rate in Switzerland, the limitations of this study (see below), and the 5-year research window. We are unable to add or draw conclusions nor improve standards because of the limited information available at our relevant institutions. To the best of our knowledge, this study is the first to provide information on ambulance accidents in Austria, Germany, and Switzerland, the three largest German-speaking countries in Europe [25]. This is also the first media-based study to collect information about ambulance crashes in these three countries.

### Comparison Between Ambulance Accidents and Overall Road Traffic Fatalities

The International Road Safety Annual Report 2019 reported that the fatality rate from RTAs was 2.7 per 100,000 inhabitants in Switzerland, 3.8 in Germany, and 4.7 in Austria [26,27]. Switzerland represents one of the best outcomes, with under 0.4 deaths per 10,000 cars owned.

According to our data, the incidence of fatal ambulance accidents per 100,000 inhabitants was 0.034 for Germany, 0.068 for Austria, but zero for Switzerland (Table 2). These values correspond to 0.97%, 1.68%, and 0% of total RTAs for Germany, Austria, and Switzerland, respectively. Since our search was restricted to free-access newspapers, the true ambulance accident death rate is probably higher. In each of these three countries, the incidence of ambulance accidents and total RTAs have both tended to decrease in recent years.

The International Road Traffic and Accident Database established that pedestrians and cyclists are most likely to suffer the fatal consequences of an RTA [26]. This is concurrent with ambulance accident injuries and fatalities in the three countries analyzed in this study (Table 2).

### Injuries From Ambulance Accidents

For each accident with an ambulance, a mean of nearly two people were injured (Germany, 1.96; Austria, 1.85), except in Switzerland where a rate of 0.72 injured per accident was reported (Table 2). In 1987, Auerbach [24] established that 2.24 injuries occurred per accident in Tennessee. Despite modernization of ambulances, more than 30 years later, these statistics remain very similar. This could also be because minor accidents with ambulances were not reported in the newspapers accessed for this study. Additionally, ambulance design and safety measures for passengers have perhaps not been sufficiently adapted for high-speed driving.

### Circumstances of Ambulance Accidents

The data suggest that in Germany and Austria, respectively 39% and 27% of ambulance accidents at intersections happened when a traffic light signaled red for the ambulance. In Switzerland, one accident (4%) took place at a red traffic light. This could be explained by available traffic signal preemption installed in emergency vehicles, which is in force in Switzerland at major intersections to allow ambulances the right of way.

In Austria and Switzerland, the media reported the cause of the accident in over 80% of the ambulance accidents, which then allowed us to categorize the cause of the accidents. The ambulance was implicated in more than half of the reported accidents in all three countries. This validates the need for further research to mitigate ambulance accident rates and the sequelae thereof. Studies have often focused on the analysis of accident prevention by the emergency service provider and institutions through ambulance engineering, driver experience/training, and environmental factors [6-9,28,29].

### Blue Lights and Sirens

Both blue lights and sirens were used in approximately 45% of the ambulance crashes in Germany and Switzerland and in 13% of these accidents in Austria. The reason for the lower use in Austria was not quite clear. In other European countries such as the Netherlands, the use of blue lights and sirens is restricted. In Switzerland, injuries occurred in 70% of ambulance accidents where blue lights and sirens were used, which is similar to the values for Germany (77%) and Austria (100%). In Germany, three people died when blue lights and sirens were used. Blue lights and sirens also can be used intermittently, and these signals are typically turned on when priority is needed in difficult traffic scenarios or when high-speed driving is deemed necessary. Sanddal et al [9] reported a 100% injury rate when an ambulance accident occurred while the blue lights and sirens were being used. Lights and sirens often cause a distraction and decrease the auditory awareness of all those who hear the signal, as people then search for the location of the emergency vehicle. An example was an incident when a police car and ambulance were coming from opposite directions and then collided at an intersection; neither vehicle heard the other, because both were driving with their blue light and sirens to the same accident. In 2017, Alexander Stevens, a German lawyer and paramedic, used “Ambulance Accidents–when speeding life savers become a risk” as the theme of his thesis. Stevens concluded that with blue light and sirens, emergency service vehicles were 4 times more likely to cause fatalities, 8 times more likely to cause serious injury, and resulted in a 17 times higher incidence of third-party trauma. Stevens’ opinion was that neither the police nor the government recorded how often ambulances were associated with an accident. He also added that the regulations for the activation of blue light and/or sirens were nebulous [30]. However, other studies have found that the higher incidence of ambulance collisions with blue lights and sirens was not statistically significant [31-33]. Missikpode et al [5] did not find an increased risk of accidents when blue lights and sirens were used. Ho and Casey [34] reported that the 3 minutes saved with blue lights and sirens was statistically significant. Additionally, Petzäll et al [35] illustrated that ambulance response time with blue lights and sirens was only 2.9 minutes shorter on urban roads and 8.9 minutes shorter in rural areas. Marques et al [36] established a mean time saved of 2.62 minutes (ranging from 26 minutes faster to 24 minutes slower) and suggested that blue lights and sirens should only be implemented where hospital intervention is required. Saunders and Heye [33] did find that the incidence of injury was higher when blue lights and sirens were used, which then vitiated the time saved. Sandal et al [9] advised the nonuse of blue lights and sirens and policies including use of these signals. It therefore appears that using blue lights and sirens significantly increases the likelihood of ambulance-related trauma, with no discernible or measurable benefit to patient outcome.

### Comparison With Other Studies

Reporter Kerstin Herrnkind quoted the only study by the German State Traffic Department reporting on ambulance accidents at the time (2017), which suggested that one fatality had occurred every 5 days due to ambulance accidents [16,17]. The 31 fatalities in Germany over the 5-year period of this study certainly represents a dramatic decrease from the study performed 10 years previously (Table 2). Herrnkind also mentioned the financial impact of these collisions.

A review of the literature for the United States showed that 19% of the fatalities involved patients being transported by ambulance at the time of the accident, 14% were staff, and 67% involved a third party [9]. In this study, we found that in Germany and Austria, 28% of the fatalities were patients, 14% were staff, and 56% a third party. In Austria, one of the two patients was accidentally run over as the ambulance reversed at the accident site.

We collected a wide range of web-based information, which allowed us to allocate accidents to each federal state in Austria and Germany, as well as to specific cantons in Switzerland, and these details could be compared with published international research. As in other countries, the time of the year and the weather did not clearly influence the incidence or occurrence of an ambulance accident [6-8,31,37,38]. However, in contrast to some other studies, we were able to distinguish the day of the week when the accidents occurred. Accidents were less frequent over weekends and at night, which can be explained by the reduced traffic over weekends and no rush hour congestion. By contrast, Ray and Kupas [37] found that ambulances were involved in more accidents than similar nonemergency vehicles over the weekends and at night. Kahn et al [6] also found that bystanders not in the ambulance were more likely to be injured or killed when an ambulance accident occurred. Intersections were the most dangerous place for pedestrians, cars, two-wheelers, children, and ambulances with or without blue lights and sirens (sometimes disobeying a red light) [24,31,33,37,38]. Our study reproduced these findings for Austria (but not for Germany) where mostly staff members were injured. This suggests that standard national procedures have not put sufficient focus on the safety of the staff. Reichard et al [39] noticed that injury events for emergency medical service workers were vehicle accidents in 8% of cases and assault and violence in 7% of cases.

Custalow [38] found that the risk of injury was higher at intersections. She advocated a safe slow approach, ensuring that other parties have stopped and maintaining eye contact at intersections where visibility may be relatively reduced in cities. Seatbelt use in the rear of the ambulance and insecurely fastened equipment were implicated in injuries to staff [23,27].

A psychiatric patient created a hazardous situation on the way to the hospital that resulted in two staff members being injured and the public endangered. The patient was unaccompanied and their behavior was unexpected. Media representation of events is not always a factually unbiased representation of true events. Cuijpers and Brown [40] have analyzed the representation of systemic and symbolic violence toward ambulance personnel.

### Data Collection and the Internet

Real-time use of Google News and other news platforms may increase the accuracy and validity of data collected. Google News and some online newspapers offer an archive of the preceding year only. Consequently, we were not able to enhance our data collection by using Google News. For Switzerland, Google News was limited to the French and German regions. Google Trends provided search-trend information for Germany only and the German word “krankenwagen” (ambulance), because too few searches were conducted on this subject in Austria and Switzerland. Peaks were observed in November 2015, 2016, and 2018, which presented no correlation to the incidence of ambulance accidents as reported in the daily online newspapers.

Social media (Twitter feeds, chatrooms, LinkedIn, WhatsApp groups, blogs) may broaden data sources on ambulance accidents and increase awareness [41]. Recruitment of participants for medical research is omnipresent and some research groups have found social media to be helpful in recruitment for their studies [42]. The use of platform strategies (homebases, embassies, and listening posts) can assist knowledge translation and dissemination. An important aspect is that Twitter hashtags (words or phrases prefixed with “#”), word clouds, and visual abstracts particular to each topic may be created to engage and connect similar-minded researchers, experts, and coaches to enhance the quality of the research and analysis performed [41,43]. A word cloud is a visual representation of the word frequency in a given text. Word cloud creation may also help find and validate the search terms and results. Before standard traditional content analysis is used, word clouds may help researchers achieve a quick and basic understanding of the resulting data [44]. Realistic caution and suspicion about the quality of online resources is necessary if professional standards are to be maintained within conducted research [43].

### Limitations

The first and probably most obvious limitation of this study is that not all newspapers were searched, as we accessed free web-based newspapers only. Notwithstanding the unknown proportion of missed ambulance accidents, we assume that this is low, as the daily regional free newspapers would report these newsworthy (sometimes sensational) ambulance accidents. Conversely, and yet for the same reason, minor ambulance accidents could be underreported.

Additionally, information is not very specific or uniform, and incidents are often reported by nonclinical, nonresearch personnel. Therefore, we cannot accurately reflect the true incidence, causes of these accidents, nor the cause of injury or death in Austria, Germany, and Switzerland. No causal, contributing, or interrelated factors could be isolated or interpreted from the newspaper articles. Several factors that have been shown to increase the risk of death and injury, such as whether the injured person was in the front or back of the ambulance, ambulance speed, human factors, and use of a seatbelt in the front and back of the ambulance (less relevant today), could not be assigned to each injured person, fatality, or even the ambulance accident itself [2,6,45]. Taken together, this limits the thoroughness and quality of the information for policy creation, changes, or their implementation.

The location of the accident was not subdivided into rural and urban ambulance accidents, since this distinction is not clearly defined in the literature at present [7,37].

The World Press Freedom Index 2020 ranks Switzerland, Germany, and Austria in 8th, 11th, and 18th position in the world, respectively, regarding the liberty of expression in the press. However, research still needs to be undertaken to study how representative the media is of the true population for ambulance accidents in these countries.

Furthermore, we were unable to compare the ambulance accident data collected from this media-based review with the information collected by local, regional, or national institutions in these countries. Such information is not freely available and special permission would be necessary to access and analyze such data. In this light, we could not calculate the incidence of ambulance accidents per total number of ambulance callouts, since the information is not easily obtainable from the relevant institutions. Nonetheless, we did not actively seek access to national protocols, safety directives, or the rights of emergency vehicles with or without blue lights and sirens to effectively interpret and compare with the media-based information obtained for Austria, Germany, and Switzerland.

Illustrations often accompany an article covering an ambulance accident. We only used the written article and annotations to the image for data collection. Photographs can provide additional data (eg, time of day, weather, location, whether blue lights and sirens were used). Despite this, the timing, size, and field covered by the photograph can be misleading and the extrapolated data may be incorrect.

Long-term sequelae and costs related to the ambulance accident reports were not investigated, because this information may not be available or easy to find in the media.

### Conclusion

An ambulance accident delays the definitive treatment of a patient, can exacerbate or cause further injury, and proves to be an expensive outcome. Additionally, such accidents compromise trust in public safety. However, despite the reports of such incidents in the media, public trust appears to remain intact. This raises the question as to whether the public has a choice. Neither the public nor law enforcement insists on further investigation or improvement in the safety of all parties involved. The numbers are relatively small and perhaps not of consequence among other causes of deaths and injuries; nevertheless, the incidence can certainly be decreased. Contributing factors have not been optimally researched and analyzed in German-speaking countries. Goal-directed controls and protocols to decrease or even avoid accidents and their costly repercussions are desperately needed.

The internet, the media, and social media together present medical research with many possibilities such as data collection, telemedicine, open-access peer-reviewed journals, and online education, along with the rapid evolution of artificial intelligence. Many accidents are already prevented by advanced driver-assistance systems, traffic signal preemption by emergency vehicles, protocols, driver training, and traffic rules signposted to guide motorists when emergency vehicles with blue lights and sirens are in proximity. Intelligent vehicle signal communication is implemented in Austria, Germany, and Switzerland. In the future, artificial intelligence may be able to predict and, alongside simulation training, help to decrease the incidence of ambulance accidents. In Europe, since 2018, eCall112 is a mandatory vehicle sensor installation, which activates an automatic call initiating an audio channel between the vehicle and the most appropriate emergency center. Perhaps eCall112 should be installed in all ambulances and the details stored in a specific database only for ambulance accidents. Smartphone apps may now be used to collect information from people present at the time of an ambulance accident and educate the general public with updated real-time use of basic life support.

Further information available in the media through apps and telemedicine can also be used to decrease the urgent nature of an ambulance call, thus decreasing the morbidity and mortality associated with ambulance accidents. However, this can only be achieved through governance of the vastly increasing online information and platforms.

In Germany, Austria, and Switzerland, ambulance drivers are accountable for damages, injury, or death caused by nonadherence to traffic regulations when responding to an emergency call. This is not always equitable. The data collection and analysis in this study may seem insufficient, but nevertheless directs us to auxiliary data sources that may allow for the creation of up-to-date registers of all ambulance accidents in Austria, Germany, and Switzerland. To improve risk management and establish European standards, it must be mandatory to collect standardized and accurate representative information using a variety of sources (press and modern strategies such as social media platforms, blogs, and targeted news groups). The control of the information concerning the accuracy of the data needs to be further researched before such analyses are performed and subsequent strategies are applied in practice. As a next step, this information should be made available for audit, analysis, and research purposes. Future research should analyze the array of human, engineering, environmental, organizational, and political factors that impact the balance between the outcome and safety of the patient, the staff, and the people present at the perimeter of an ambulance call.

## Abbreviations

AAES: Austrian Ambulance Emergency Services
RTA: road traffic accident
